# Media Representations of Science during the First Wave of the COVID-19 Pandemic: A Qualitative Analysis of News and Social Media on the Island of Ireland

**DOI:** 10.3390/ijerph18189542

**Published:** 2021-09-10

**Authors:** Cliodhna O’Connor, Nicola O’Connell, Emma Burke, Ann Nolan, Martin Dempster, Christopher D. Graham, Gail Nicolson, Joseph Barry, Gabriel Scally, Philip Crowley, Lina Zgaga, Luke Mather, Catherine D. Darker

**Affiliations:** 1 School of Psychology, University College Dublin, D04 Dublin, Ireland; 2 Discipline of Public Health and Primary Care, Institute of Population Health, Trinity College Dublin, D02 Dublin, Ireland; oconnen4@tcd.ie (N.O.); burkee16@tcd.ie (E.B.); nicolsg@tcd.ie (G.N.); joebarry@tcd.ie (J.B.); zgagal@tcd.ie (L.Z.); lmather@tcd.ie (L.M.); catherine.darker@tcd.ie (C.D.D.); 3 Trinity Centre for Global Health, Trinity College Dublin, D02 Dublin, Ireland; nolana13@tcd.ie; 4 School of Psychology, Queen’s University Belfast, Belfast BT9 5BN, UK; m.dempster@qub.ac.uk (M.D.); christopher.graham@qub.ac.uk (C.D.G.); 5 School of Medicine, University of Bristol, Bristol BS8 1QU, UK; gabriel.scally@btinternet.com; 6 Quality Improvement, Health Service Executive, D08 Dublin, Ireland; philip.crowley@hse.ie

**Keywords:** COVID-19, coronavirus, pandemic, science, news media, newspapers, social media, Twitter, qualitative

## Abstract

COVID-19 is arguably the most critical science communication challenge of a generation, yet comes in the wake of a purported populist turn against scientific expertise in western societies. This study advances understanding of science–society relations during the COVID-19 pandemic by analysing how science was represented in news and social media coverage of COVID-19 on the island of Ireland. Thematic analysis was performed on a dataset comprising 952 news articles and 603 tweets published between 1 January and 31 May 2020. Three themes characterised the range of meanings attached to science: ‘Defining science: Its subjects, practice and process’, ‘Relating to science: Between veneration and suspicion’ and ‘Using science: As solution, policy and rhetoric’. The analysis suggested that the COVID-19 pandemic represented a platform to highlight the value, philosophy, process and day-to-day activity of scientific research. However, the study also identified risks the pandemic might pose to science communication, including feeding public alienation by disparaging lay understandings, reinforcing stereotypical images of scientists, and amplifying the politicisation of scientific statements.

## 1. Introduction

Understanding the COVID-19 pandemic’s cultural legacy requires interrogation of the specific impacts that the virus and its countermeasures have had in all societal domains. Unlike the many cultural and economic institutions that public health restrictions have immobilised, some scientific fields have seen an unprecedented boost in their public profile and funding. With individual and societal recovery resting on scientific advances in understanding, preventing and treating COVID-19, the pandemic has underscored the existential importance of scientific activity. Yet the pandemic has also publicly exposed instances of flawed or fraudulent research, dissension within the scientific community, misdirected policy advice, and appropriation of scientific claims to serve politicised interests [1]. The ways the pandemic has affected science–society relations, which in western societies were already under challenge from a purported cultural turn against expertise [2], remain to be seen. The current paper reports a qualitative analysis of how science was represented in news and social media during the early months of the COVID-19 pandemic on the island of Ireland. By inductively exploring the meanings attached to the term ‘science’ in popular media, the analysis aims to reveal how public discourse defined, interpreted and evaluated science during the pandemic.

### 1.1. The Science-Society Relationship

Several social theorists have characterised public responses to science as profoundly ambivalent [3,4,5]. The demise of religious influence in post-industrial Western societies allowed science to forge a monopoly on the production of credible knowledge. However, Habermas [5] contends that the resultant proclivity for tackling social problems through technocratic solutions undermined opportunities for democratic public participation in decision-making, feeding an ‘institutional alienation’ in which sectors of the public feel socially detached from scientific elites. This public alienation from science is further elaborated by sociologists Beck [3] and Giddens [4], who observe that while technological innovation is a crucial motor of social progress, it has also produced many human hazards, such as environmental pollution, nuclear disasters, and antibiotic-resistant infectious diseases. Disenchantment from science has recently become a growing focus of political discourse worldwide. Public support for populist causes (e.g., Brexit in the UK) or leaders (e.g., Donald Trump in the US or Jair Bolsonaro in Brazil) has been interpreted as reflecting antipathy towards experts, who are equated with elitism and intellectual arrogance [2,6,7,8]. This cultural moment was exemplified in the selection of “post-truth” as the 2016 Oxford Dictionaries word of the year, connoting a rise of “circumstances in which objective facts are less influential in shaping public opinion than appeals to emotion and personal belief” [9].

The ambivalent quality of the public’s relationship with science is borne out by empirical research suggesting lay attitudes to science are complex and contradictory. On the one hand, overt support of the scientific community is high. For example, a 2019 survey of 22 countries identified scientists as the world’s most trusted profession [10]. In the UK, annual surveys show the proportion of the population who trust scientists is increasing, rising from 63% in 1997 to 84% in 2019 [11]. However, this trust is tempered by pockets of unease with scientific activity. Three-quarters of UK respondents believe funders compromise scientific independence and approximately half believe scientists are “secretive”, invest too little effort in communicating with the public, and engage in unregulated activity “behind closed doors” [12]. Thus, despite globally positive inclinations towards science, sizable portions of the public express reservations that intimate a sense of suspicion.

Within the scientific community, trust in other scientists’ findings is sustained by understanding the processes that produce and audit scientific work, such as peer review and conflict-of-interest disclosures [13]. However, most lay publics are less familiar with research processes [14], so rely on other information to infer scientific trustworthiness. Public trust in science can be especially affected by events that highlight the risks or shortcomings of scientific knowledge. For example, the 2011 Fukushima nuclear accident had lasting repercussions for public trust in science in Japan [15,16]. As the most high-profile science story of a generation [17], it is similarly possible that science’s inability to prevent the death and destruction wreaked by COVID-19 may weaken public trust [18]. Research indicates that people who were exposed to local pandemics in early adulthood show lower lifetime confidence in science [19]. Yet it is also plausible that the pandemic will bolster public support for science by illuminating human populations’ existential dependence on scientific developments (such as vaccines). In February 2020, 69% of US survey respondents stated that responses to the approaching COVID-19 pandemic should be led by scientists, with only 14% ascribing leadership roles to politicians [20]. A survey of 2,237 UK residents in July 2020 found 49% stated their trust in scientific experts had increased due to their work on the COVID-19 response [21]. However, other research from both the UK and the US finds no discernible change in scientific trust during the pandemic [22,23]. Indeed, one UK poll suggests the proportion characterising scientists as trustworthy slightly decreased during the early months of the pandemic, from 62% in April to 49% in May [23].

To date, most research on public orientations to science during the COVID-19 pandemic has emanated from the UK and US—countries which have recorded among the highest deaths per capita internationally to date [24]. Broadening of research contexts is needed to fully understand societal responses to this global pandemic. The current paper focuses on the island of Ireland. While the Republic of Ireland (ROI)’s COVID-19 mortality rates have been among the lowest in Europe, Northern Ireland (NI), a jurisdiction of the UK, has recorded higher per capita case and death rates [25]. Little concrete data illuminate public attitudes to science in NI. However, ROI entered the pandemic with high baseline support for science: despite spending below the EU average on research [26], Irish residents rank above the EU average in public knowledge and interest in science and have the EU’s second-highest levels of optimism about science’s societal role [27]. In 2015, 22% of the population had ‘a lot’ of trust and 60% ‘some’ trust in scientific advice, with medical discoveries the category of science attracting most public interest [27]. A mid-pandemic survey in May 2020 showed scientists ranked with healthcare professionals among the most trusted sectors of society: 87% trusted scientists to tell the truth, a slight increase from 2018 [28]. In the same survey, 91% of ROI residents reported high trust in the National Public Health Emergency Team (NPHET), the body providing medical and scientific advice on COVID-19 to the Irish government [28]. This compares favourably with equivalent UK data, where 55% reported trusting the scientists advising the government’s COVID-19 response [23]. However, some pre-pandemic data indicate Irish residents may harbour reservations towards certain aspects of COVID-related science: a 2015 survey found just 27% reported they would have ‘a lot’ of trust in a scientific statement about vaccines [27]. Additionally, the two years prior to the pandemic saw extensive media coverage of a scandal involving the national cervical screening programme, which focused public attention on the risk of misleading (false negative/false positive) laboratory tests and the quality of laboratory services [29]. As this context of globally positive scientific attitudes coexisting with specific scientific controversies resembles many countries worldwide [10], Ireland may prove a useful case study to explore how lay publics are assimilating the heightened attention to science brought by COVID-19.

### 1.2. Science in the Media

COVID-19 has generated unprecedented levels of coverage in both news and social media internationally [30]. This media content offers a valuable platform for understanding public engagement with science during the pandemic. As only small pockets of the population come directly into contact with ‘pure’ science, the media are the primary vessel by which scientific ideas are transmitted to public awareness [31]. Media do not simply insert information into a ‘blank slate’ of public consciousness: audience reception is a constructive process, which interprets information through the prism of pre-existing values, identities and beliefs [32,33,34]. However, the media can cultivate particular ideas in their audience by selectively directing attention towards specific applications or controversies [35]. For example, media coverage that linked positions on climate change with specific political affiliations was instrumental in shifting public opinion over time [36]. Thus, the content of media coverage can both shape and reflect the sociocultural meanings that a scientific topic assumes as it moves into public consciousness.

In recent years, shifting patterns of media consumption have seen increasing delivery of scientific information via online sources such as social media [37]. Concerns about the rise of a ‘post-truth’ public sphere often implicate social media platforms, which increase the circulation of information produced by vested interests without quality- or fact-checking [2,8]. Surveys during the pandemic indicate people desired more information about COVID-19 and actively sought information via diverse sources [23]. A UK survey found 33% accessed scientific information on COVID-19 via newspapers and 27% on social media, with social media use rising to 44% in the 16–24 age group [23]. Different media sources show variable relationships with COVID-19 protective behaviours, which (controlling for age and gender) are negatively associated with social media use but positively correlated with traditional media consumption [38].

Some literature suggests that a suppressive effect of social media on public health behaviours may be due to exposure to ‘misinformation’, i.e., posts belittling the severity of the virus or effectiveness of behavioural containment measures. In previous infectious disease outbreaks social media networks, such as Twitter, have been implicated in the spread of misinformation [39,40]. A recent study found a reliance on social media for COVID-19 news predicted increased endorsement of COVID-19 ‘conspiracy theories’, which in turn correlated with reduced protective behaviours [38]. Research indicates that people are selectively influenced by online scientific information that supports pre-existing attitudes [41] and that falsehoods diffuse more rapidly and widely than truthful statements on Twitter [42]. However, recent coronavirus-specific research has found the reverse, with science-based tweets retweeted more than false information [43] and posts from ‘reliable’ news sources vastly outnumbering those from ‘questionable’ sources on mainstream social media including Twitter [44]. Thus, the COVID-19 pandemic may be producing distinct social media dynamics that depart from previously observed patterns. For example, perhaps the gravity of the pandemic, combined with efforts from social media providers to flag and curb content deemed misinformation [45], has motivated more screening and sharing of trustworthy information.

The few studies of social media communication about COVID-19 published to date predominantly focus on the veracity of social media content [44,46,47,48,49]. This is asynchronous with broader trends in social studies of science, which have largely rejected the utility of ‘deficit models’ of science communication, i.e., approaches that focus primarily on evaluating the *accuracy* of lay understandings of scientific information [50]. Deficit models have been challenged on grounds of the contingent, changeable nature of scientific knowledge (for instance, scientific advice on face-coverings has changed during the pandemic [51,52]), and evidence that lay deviation from scientific consensus can reflect adaptive, ecologically rational knowledge rather than error [53,54]. Moreover, focusing purely on the accuracy of lay understandings offers limited utility for understanding public support of science since the relationship between public knowledge and attitudes is not linear but U-shaped: the most negative attitudes to science are concentrated among those with highest scientific literacy [50,55]. As such, understanding public orientations to science during the pandemic may be best served not by arbitrating the factual accuracy of media communications, but analysing how communities make sense of new information by relating it to prevailing networks of cultural values, beliefs and ideologies [32]. Qualitative research is ideally suited to exploring the sociocultural meanings that a scientific topic appropriates [32]. To date, minimal qualitative research has examined the roles and meanings attributed to science in media communication about the COVID-19 pandemic.

### 1.3. The Current Study

The current study explores media representations of science in the early months of the COVID-19 pandemic on the island of Ireland using two sources: news articles and Twitter posts (tweets). This study forms part of a more extensive interdisciplinary project [56] examining the psychological, behavioural, media and governmental responses to COVID-19 in Ireland. Previous evidence indicates newspapers and social media are among the sources most used by Irish residents when searching for scientific information [27]. While numerous studies of COVID-19 on social media are emerging internationally [44,46,47,48,49], very little research investigates COVID-19 coverage in news media [57], despite surveys indicating news media are people’s primary source of COVID-19 information [23]. While newspaper readership is declining, 4-in-5 Irish adults read newspapers weekly before COVID-19 [58], with over a quarter stating their newspaper consumption increased during the pandemic’s early months [59]. It is worth noting that national newspapers in Ireland are not polarised along traditional left/right dimensions and do not openly identify with a particular political ideology [60], although some NI newspapers are traditionally affiliated with either a Unionist or Nationalist perspective on the NI conflict.

Irish social media use also increased during the pandemic [59]. Relative to other European countries, ROI residents rely more on online sources of scientific information [27]. Twitter is among the most popular social networking sites in Ireland, used by 30% of the ROI population [61], and was chosen for this study due to its accessibility for research purposes.

The study applies thematic analysis to identify the range of meanings attached to references to science in both news and social media. The datasets encompass the period from 1 January to 31 May 2020, which captures initial reporting of the outbreak in Wuhan, China; the first confirmed case of COVID-19 on the island of Ireland (27 February); and the ‘first wave’ of the pandemic in Ireland, which peaked in mid-April with over 1000 daily cases and over 70 daily deaths. The analysis includes media content from both ROI and NI due to the extensive interpenetration of media across the island’s invisible land border. While distinct scientific advisory groups informed the jurisdictions’ pandemic responses, which at times implemented diverging policy measures [25,62], this facilitates an interesting exploration of how public responses to science may be affected by exposure to differing scientific advice on the one landmass. The study does not predefine the specific scientific fields under analysis but instead explores how the category of ‘science’ was construed, interpreted and applied in popular usage. The exploratory analysis is guided by the research question: how did Irish news and social media represent science during the ‘first wave’ of the COVID-19 pandemic?

## 2. Materials and Methods

### 2.1. Data Collection

News articles were harvested from the Nexis Advance database. Boolean searches identified articles published between 1 January 20 and 31 May 20, which contained the keywords “scien*” and “COVID* OR corona* OR SARS-Cov-2”. The search identified 952 articles from 11 newspapers with a publication location on the island of Ireland. Table 1 presents the number of articles that came from each newspaper. Full texts of all articles were downloaded for analysis.

Twitter content was acquired from a media analytics service (Vicinitas). To devise an appropriate data extraction strategy, exploratory searches of the Twitter interface identified relevant terms and hashtags used in the Irish context. The dataset comprised tweets geolocated to the island of Ireland and published between 1 January 2020 and 31 May 2020, which contained the words or hashtags “COVID OR COVID19 OR COVID-19 OR COVID_19 OR coronavirus OR corona OR COVID19ireland OR coronavirusireland OR COVID19northernIreland OR COVID19NI OR COVID19UK”. Additional searching within this dataset identified tweets that contained the word-stem “scien*”. A spreadsheet containing the content of 603 tweets (Table 2) was extracted for analysis. Due to ethical and data protection restrictions, usernames and other identifying metadata were excluded from the dataset.

The Twitter and newspaper data were collected independently and had minimal redundancy in their content; only 20 (3.3%) of tweets included a link to an article from one of the newspapers included in the analysis. 

### 2.2. Data Analysis

All data were imported into NVivo 12 for thematic analysis [63]. Extensive reading of the data informed inductive development of a coding-frame that reflected recurrent patterns of meaning. A common coding-frame was applied to both news and social media to facilitate a holistic analysis of public discourse. The coding-frame grouped basic codes into broader categories to ensure accessible coding (for instance, the category *‘Expertise’* included the codes *’Respect for experts’*, ‘*Expertise questioned*’ and *’Non-experts dismissed’*). The coding frame was entered into NVivo so that data units could be electronically ‘tagged’ with relevant codes. Each article/tweet was first coded by a single coder, who could apply multiple codes to an article/tweet if relevant. While formal inter-coder reliability statistics were not computed, all coding was reviewed by a second experienced qualitative analyst, with disagreements resolved through team discussion. Additional steps taken to assure the quality of the analysis included maintenance of a detailed ’audit trail’, transparent reporting of analytic procedures, ensuring all analytic conclusions were supported by illustrative data excerpts, and attention to discrepant cases [64].

Once coding was finalised, NVivo’s Query, Crosstab and Matrix functions were used to explore links between codes. These links informed the gradual development of themes and subthemes that reflected the range of meanings ascribed to science. The final thematic structure comprised eight subthemes, assembled into three core overarching themes: ‘*Defining science: Its subjects, practice and process*’, ‘*Relating to science: Between veneration and suspicion*’, and ‘*Using science: As solution, policy and rhetoric*’. The content of each is outlined below with illustrative quotes. In this qualitative thematic analysis, the priority is interpretative analysis of the range of meanings within the data, rather than quantifying code prevalence [65]. By including multiple data sources, the intent is not to contrast news and social media content, but rather to identify prevailing trends across the various layers of public discourse.

### 2.3. Ethical Considerations

Ethical approval was granted by the National Research Ethics Committee for COVID-19 research (ref. 20-NREC-COV-037). Ethical and data protection guidelines required exclusion of Twitter usernames or handles, making it impossible to obtain informed consent from tweet authors. Social media research guidelines recommend that when informed consent is not possible, quoted social media content should be slightly paraphrased in publications to minimise the risk of identifying original authors [66]. The quoted tweets below retain the meaning and tone of raw data, but have undergone minor editing (e.g., re-ordering clauses, substituting synonyms). News articles are a matter of public record and quoted verbatim.

## 3. Results

### 3.1. Theme 1. Defining Science: Its Subjects, Practice and Process

This theme captures how the media framed the topics studied by scientists, as well as reports of the day-to-day activity of scientific research, and accounts of the philosophy and epistemology of the scientific process.

#### 3.1.1. The Subjects of Science

The emergence of the novel coronavirus prompted specific attention to certain scientific fields, notably epidemiology and immunology. Results of epidemiological modelling studies were widely covered in news media, framed as either optimistic or pessimistic predictions of the near future.

*A Nobel prizewinning scientist has predicted, through analysing raw data, that Ireland’s death rate and infection will “burn itself out” in the next two weeks, enabling an earlier exit from lockdown*.

(*Sunday Independent*, 31 May 2020)

*The single most stark scientific indicator of the wrecking ball that’s going to hit was the result of modelling by a team at Imperial College London. It predicted a massive overload of hospitals and many deaths*.

(*Irish Times*, 23 March 2020)

In the early stages of the pandemic in particular, news media carried ‘explainers’ of key unfamiliar scientific concepts. As the crisis developed, such technical concepts began to be mentioned without contextualising definitions, indicating media presumption of audience understanding.

*Over the past few weeks, scientists have built mathematical models to predict what will happen with COVID-19. For the scientists, one of the key figures is the Reproduction number of a disease. This is known as R*.

(*Irish Independent*, 21 March 2020)

Juxtaposed against media coverage of highly specialist scientific concepts was the notably non-technical nature of the core public health message. In the months studied, the sole means of infection control were not sophisticated medical interventions implemented by trained experts, but lay citizens’ adherence to hand-washing, physical distancing and face-covering. The relatively comprehensible rationale for such practices and their alignment with ‘common sense’ or folk wisdom was invoked as an advantage in public health messaging.

*[…] techniques for dealing with pandemics have changed little since the 19th century. Surgical masks are now high-tech, but old fashioned hand-washing is still the core technology for preventing contagion. And by far the most effective way to limit the spread of a virus is to physically isolate all people who may be infected*.

(*Irish Independent*, 6 February 2020)

*Scientists’ breakthrough in fighting #coronavirus involves the old wives’ tale that says everyone should carry a handkerchief. Coughs and sneezes spread diseases*…

(*Tweet*, 1 February 2020)

The foregrounding of public behaviour redirected media interest from biomedical to behavioural science.

*[…] the capacity of the Government, the State and the people to manage this crisis is rooted in the science of how people behave*.

(*Irish Times*, 19 March 2020)

However, while research on human behaviour was included within the designation of ‘science’, some doubt regarding the legitimacy of such expertise remained.

*Critics angrily demanded that ministers listen only to medical and scientific experts—the sort of people who know how viruses behave and how to stop them—and that was understandable enough. Most psychology is just made-up nonsense, as the more honest ones in the profession privately admit*.

(*Belfast Telegraph*, 27 April 2020)

*On TV debates all economists are now COVID experts*.

(*Tweet*, 23 March 2020)

#### 3.1.2. The Practice of Science

The pandemic proved a platform for publicising details about the day-to-day practice of scientific research. The news media showed particular interest in established procedures of vaccine development, and potential changes to these procedures with COVID-19.

*Scientists are attempting to find ethically acceptable ways of speeding up timelines. Some are considering unconventional ways to speed up the process by injecting healthy adults with live coronavirus. Normally, it would never be considered to test a deadly virus for which there is no cure, but these are extraordinary times*.

(*Irish Times*, 30 May 2020)

The focus on vaccines, along with possible treatments for COVID-19, brought attention to the role of industry in funding and conducting scientific research. News media coverage of pharmaceutical companies’ research was often presented within a framework of commercial competition.

*It’s the holy grail jab that the world is waiting for. But just how long will it take to produce a safe and effective coronavirus vaccine which can be licensed for global use? There are two races going on at the same time. One is among pharmaceutical companies to produce the first vaccine, and the other is to have a jab that will outrun the spread of the virus*.

(*Irish Independent*, 10 April 2020)

There was both praise and criticism of privatised interests in research and development in the context of a global pandemic.

*In responding to the pandemic, the global scientific community has shown a remarkable willingness to share knowledge of potential treatments, coordinate clinical trials, develop new models transparently, and publish findings immediately. In this new climate of cooperation, it is easy to forget that commercial pharmaceutical companies have for decades been privatising and locking up the knowledge commons by extending control over life-saving drugs through unwarranted, frivolous, or secondary patents, and by lobbying against the approval and production of generics. With the arrival of COVID-19, it is obvious such monopolisation comes at the cost of human lives*.

(*Irish Examiner*, 28 April 2020)

Alongside industry, the pandemic drew attention to another subset of scientific actors: the staff in laboratories who process diagnostic tests.

*Medical scientists play a vital role in quality laboratory diagnostics for COVID-19. They are the unsung heroes*.

(*Tweet*, 5 March 2020)

*Medical scientists working in laboratories across the country are among the unseen heroes working in the background of the COVID-19 response*.

(*Irish Examiner*, 11 May 2020)

#### 3.1.3. The Process of Science

The months analysed contained real-time coverage of emergent research on a novel virus. Scientific advocates grasped this opportunity to reinforce public understanding of the scientific method and philosophy of science.

*[…] sustaining public trust in science and scientists will require us talking about how science is done, as well as the outcomes of its work. The ways in which scientists reach consensus are as important as the findings: process is at the core of our expertise. […] we must make the scientific process more explicit in how we communicate science. In its most basic form, the scientific process is built on experimentation; scientists collect evidence which may or may not confirm hypotheses. This cautious process does not result in absolute certitude. Rather, it quotes findings with error bars. It generalises with caution. It is open and revisionist. Embracing uncertainty, questioning reliability and foregrounding the hypothetical, this process gives us varying degrees of confidence in what we might say*.

(*Irish Times*, 14 May 2020)

The provisional nature of scientific understanding, which is necessarily subject to disconfirmation with new evidence, was emphasised in numerous media articles.

*The scientists leading the global crisis response are at pains to stress the limitations and the volatility of their projections. A reassuring aspect of the Irish response has been the willingness of the National Public Health Emergency Team, led by chief medical officer Tony Holohan, to admit to mistakes—as they did when they prematurely widened testing eligibility criteria. Politicians are less comfortable admitting to their limitations; listen to how Johnson says Britain will be fine because it has “the best science”. But by over-selling our scientists’ ability to steer us out of the crisis, we jeopardise public trust in science in the future*.

(*Irish Times*, 18 April 2020)

While certain articles and tweets (often produced by scientists themselves) endorsed scientific uncertainty as indexing the integrity of the research process, others focused on its implication of dissensus within the scientific community. Exposure to conflicting scientific ideas provoked frustration and anxiety, with indications the lay public felt ill-equipped to appraise the relative credentials of competing experts.

*I’m fed up with experts—both scientists and economists—bombarding us every day with daft statistics and scare stories which are often conflicting and only add to the general sense that nobody actually has a clue. These are difficult times and we will face difficult times in future. We all acknowledge that. What’s not helping is the daily blitz of contradictory reflection, prediction and supposition. Before COVID (BC), scientists in particular would have been seen as reliable, solid sorts whose pronouncements were evidence-based and therefore pretty much indisputable. You would have assumed that generally there would be consensus because the science was there to back their analysis. But what the current crisis has shown is that scientists are given to more squabbling, feuding and back-biting than the cast of Love Island*.

(*Belfast Telegraph*, 8 May 2020)

Scientific dissensus was positioned as particularly problematic for developing scientifically-determined policy decisions

*But the scientists behind the scenes are the ones having to come up with the answers, the strategy, when there is no obvious scientific consensus. That’s the worrying bit. Every time you turn on the TV there are at least three “experts” joining us onscreen from their echoey front rooms to argue with one other. Scientists are only human but they’re being depended upon to guide decisions that could cost tens of thousands of lives*.

(*Belfast Telegraph*, 3 April 2020)

*Is the scientific advice used by Leo Varadkar and Boris Johnson conflicting? Surely scientific advice should avoid that? Why we and the southern part of this small island, are acting so differently is puzzling and scary*.

(*Tweet*, 12 March 2020)

### 3.2. Theme 2. Relating to Science: Between Veneration and Suspicion

This theme describes the range of social and emotional relationships with science, both positive and negative.

#### 3.2.1. Veneration of Science

Throughout the news and social media, there were frequent expressions of admiration of science. These hailed the collective effort of science as an institution, in speedily and effectively responding to a novel public health threat.

*The achievements of the scientific community are incredible given that this virus was first discovered only a few months ago*.

(*Tweet*, 8 March 2020)

*When the COVID-19 strain appeared, scientists were quickly able to analyse it, test for it, trace its mutations, and begin work on a vaccine. While there is still much more to learn about the new coronavirus and its effects, without science we would be completely at its mercy, and panic would have already ensued*.

(*Irish Independent*, 11 March 2020)

On social media, expressions of gratitude sometimes grouped scientists with frontline workers.

*Not all heroes wear capes, some use pipettes and finely-honed analytical skills.*

(*Tweet*, 19 March 2020)

*We are so grateful to the health professionals, researchers and scientists, who are working under huge pressure around the clock to protect us*.

(*Tweet*, 14 March 2020)

Admiration of scientific achievement was heavily personalised. The news media carried personal profiles of individual scientists, who had come to national prominence during the crisis. These individuals, such as the Republic of Ireland’s Chief Medical Officer Dr Tony Holohan, were ironically imbued with an air of celebrity.

*Is William Gerard Anthony Holohan the coolest man in Ireland right now? Everyone I know thinks so, and it seems the entire nation has come to welcome his daily intrusions into our lives as the ultimate feelgood bedtime story. The chief medical officer for the Irish Department of Health, Tony Holohan has become a shining light of hope during these dark days, someone whose assured delivery and easy style continues to sooth the shattered nerves of the nation and furnish hope that everything will be all right. Women especially display an obvious affection for him, commenting dreamily on his dulcet tones and fatherly demeanour. When he visited hospital recently for a routine check-up, the nation held its breath in similar fashion to Packie Bonner’s legendary goalkeeping at the 1990 World Cup. Transfixed ladies say ‘Tony’ with a husky timbre, paying rapt attention to his spectacles and how he carries his briefs*.

(*Irish Independent*, 13 April 2020)

Newspapers carried profiles of home-grown scientists who were working on coronavirus-related projects.

*A Fermanagh-born scientist working on a vaccine to stop the spread of coronavirus has spoken of her pride at being involved in the groundbreaking project in the United States*.

(*Impartial Reporter*, 31 May 2020)

Numerous news articles explicitly contrasted the preponderance of public support for science with a contemporary context of declining faith in expertise. The COVID-19 pandemic was positioned as sparking a re-embrace of scientific knowledge and expert authority.

*Experts were falling out of vogue. It is perhaps unsurprising that it took a global crisis—of truly unprecedented proportions—to redress this troubling phenomenon. […] for the most part, when it comes to questions of science and medicine people seem far more submissive to expertise; far less inclined to claim the authority to wax lyrical on their latest theories; and far quicker to exalt the doctors, scientists and researchers on the frontline. […] We need to listen to the experts and we don’t have time to tolerate the latest crackpot theory from the telegenic buccaneering ideologues endemic to the British media. It is a small relief in an otherwise troubling time. And while it remains to be seen whether it will have any lasting impact, the heartening resurgence of faith in our experts—though heralded by global crisis—is worth celebrating*.

(*Irish Times*, 27 March 2020)

On social media, expressions of trust in science explicitly juxtaposed it with sources deemed less reliable, notably politicians.

*I will listen to the science and not the politicians—with this virus, the scientific community is my best bet*.

(*Tweet*, 31 May 2020)

*The COVID-19 crisis highlights the value of experts, we need to trust and invest in scientists and researchers rather than populism and simplistic slogans*.

(*Tweet*, 18 March 2020)

Numerous commentators aired hopes that COVID-19 would broker more positive relationships between science and society, which would endure beyond the public health crisis to other societal challenges such as climate change.

*There is an opportunity to re-imagine our relationship with the planet and an opportunity to re-imagine our relationship with science and to understand that we should respect science whether it’s epidemiology, climate or any number of other disciplines*.

(*Irish Independent*, 18 March 2020)

However, the veneration of scientific expertise served as a vehicle for some reversion to deficit model understandings of the science–society relationship. Particularly on social media, there were repeated statements that people without scientific credentials should refrain from commenting on the coronavirus response.

*People who aren’t scientific experts should save their armchair views on how to deal with COVID-19*.

(*Tweet*, 9 March 2020)

*If you don’t do science, be modest, shut up and listen to the experts*.

(*Tweet*, 28 February 2020)

#### 3.2.2. Suspicion of Science

Direct criticism of science was rare in either news or social media. Methodological critiques of specific scientific studies were near-absent within the data: when criticism did occur, it primarily arose from frustration with dissenting scientific opinions.

*When one country takes a completely different approach than the rest of the world, it might be time to question the “brilliant” scientists these countries are using*.

(*Tweet*, 21 May 2020)

While a handful of tweets offered an image of scientists as malevolent actors, these were largely humorous or ironic in tone.

*Scientists have produced an exact laboratory replica of the coronavirus... lots of films make me think that’s a bad idea*

(*Tweet*, 29 January 2020)

A small number of tweets and news articles mooted the possibility that COVID-19 was created in a Chinese laboratory, and released either deliberately or accidentally.

*Apparently coronavirus escaped from a science lab in China that keeps diseases like Ebola to experiment with. If true, China have a lot to answer for*.

(*Tweet*, 29 February 2020)

*But this obscure story has chilling echoes today for experts who fear the current pandemic—SARS-CoV-2 is the scientific name of the new coronavirus which causes COVID-19 in humans—emerged not from Wuhan’s Huanan market, but escaped from one of the city’s two laboratories experimenting with bat coronaviruses. One of these laboratories—run by the Chinese Centre for Disease Control and Prevention—is very close to the Huanan market*.

(*Irish Daily Mail*, 18 April 2020)

However, in both datasets, the idea that the virus was lab-created appeared much more frequently in the context of articles or tweets refuting this theory.

*The US President said he had seen* *evidence that the virus came from an infectious diseases laboratory in Wuhan and suggested its release was a “mistake”. But Dr Michael Head, a senior research fellow in global health at the University of Southampton, said: “We have good evidence from the genomics research that the virus is not man-made. The scientific world has moved on from this idea. It is unhelpful for high-profile individuals to repeat the debunked conspiracy theories because it undermines the public health response.”*

(*Belfast Telegraph*, 2 May 2020)

*A great breakdown of why it’s unlikely that coronavirus originated in the Wuhan Institute of Virology, contrary to some Internet opinion. Science communication is so important right now so please share*.

(*Tweet*, 15 May 2020)

Similarly, references to the idea that COVID-19′s origins lie in 5G telecoms only occurred in the context of its refutation. Delegitimising such causal accounts was often achieved by disparaging those who endorse them and ascribing the aspersive label ‘conspiracy’.

*[…] why are others so gullible, so quick to believe the most bizarre, nutty things when surely in their heads there must be a small, nagging voice telling them: no, this can’t possibly be accurate, logical or advisable? In the case of COVID cures, all of us want to believe there’s an answer. Fear makes people clutch at straws. The 5G conspiracy theory comes from a darker place, however. And the sad fact is that there will always be people who believe such nonsense*.

(*Belfast Telegraph*, 23 May 2020)

*Public figures with huge followings should be ashamed for circulating this bogus conspiracy rumour about the origin of the virus*.

(*Tweet*, 30 April 2020)

Some data articulated concern that the pandemic had increased the scale and stakes of the spread of misinformation in society. The news media implicated social media in the dissemination of misinformation.

*Sadly, the rapid perpetuation of falsehoods has been a hallmark of the outbreak. Dubious messages circulating on WhatsApp especially have created needless panic, despite being utterly bereft of any veracity*.

(*Irish Times*, 5 March 2020)

*There are fears that Facebook’s algorithm could be “radicalising” Irish social media users, and directing them down rabbit holes which promote conspiracy theories about COVID-19*.

(*Irish Independent*, 21 May 2020)

Conversely, the social media data contained criticism of the scientific credentials of mainstream print media coverage.

*There’s an article with dangerous assumptions of immunity in the Independent with no peer reviewed studies or scientific articles checked*.

(*Tweet*, 5 April 2020)

*As a long-time subscriber to The Economist, this article lacks ANY scientific research on the link between Coronavirus survival and smoking and is circumstantial at best*.

(*Tweet*, 3 May 2020).

### 3.3. Theme 3. Using Science: As Solution, Policy and Rhetoric

The final theme captures media representations of science’s functions in society: to directly resolve the COVID-19 pandemic, inform evidence-based public policy, and serve rhetorical purposes in political discourse.

#### 3.3.1. Science as Solution

Science was frequently positioned as the only means of escape or rescue from the threat of COVID-19 and its societal disruption. In both news and social media, science was framed as a repository of hope in anxious times.

*There are plenty of other new viral candidates waiting in the wings, guts, breath and blood of animals around us, and any one of these infections, along with countless as-yet-unknown zoonoses, could cause a global disaster beyond the worst nightmares of Hollywood. Our hopes must lie, as ever, in science*.

(*Irish Daily Mail*, 27 January 2020)

*When I wake up each morning I google “Covid19 cure or vaccine”. Godspeed to all the amazing scientists*.

(*Tweet*, 23 March 2020)

The most highly anticipated scientific breakthrough was an effective vaccine. A vaccine’s consequences were framed not merely in terms of reducing morbidity and mortality, but facilitating a resumption of ‘normal life’.

*A vaccine will ultimately be the thing that will protect us and allow life to return to normal although in a different world*.

(*Sunday Independent*, 5 April 2020)

Given the hopes invested in a prospective vaccine, commentators in both news and social media worried that vaccine hesitancy among specific populations (often specifically referring to the US rather than Ireland) would curtail achievement of population immunity. However, within the datasets, no statement directly expressing vaccine rejection or reluctance was identified.

*[…] only half of Americans said they would be willing* *to get vaccinated if scientists are successful in developing a vaccine, according to a poll*

(*Irish Examiner*, 28 May 2020)

*Given much of the US public actively reject anything from experts or science, I expect numerous waves of COVID-19 in the States before this is over. Convincing people to use vaccines won’t be an easy task*.

(*Tweet*, 17 April 2020)

#### 3.3.2. Science as Policy

While society awaited COVID-19 vaccines or treatments, the only means of controlling the virus lay in constraining public behaviour. Both news and social media reflected a conception that scientific experts had a leading influence in determining specific political decisions, such as banning mass gatherings, requiring face-coverings or restricting travel.

*The previous advice was to wear a mask if you had a cough, because the cough droplets containing the virus would be trapped in the mask. But when we learnt that you can spread the virus without symptoms, and even by just speaking, it made sense for everyone to wear a mask, and it still does. The science worked: it changed our view on masks*.

(*Sunday Independent*, 24 May 2020)

*Sadly science says no to mass gatherings in 2020*.

(*Tweet*, 16 April 2020)

There was widespread explicit and tacit approval of the notion that policy should be based on scientific evidence. Science was privileged above potential alternative influences on policy decisions, such as political interests or public opinion.

*[…] the idea that something as nebulous as public opinion could be the key factor in a decision of such critical importance—one that requires the most thoroughly-researched, deeply-nuanced scientific judgments—disturbs me. […] This is no time to put our faith in the wisdom of crowds. The science may be flawed, it may lack vital data, and it may be disputed—but right now it’s all that we’ve got*.

(*Belfast Telegraph*, 16 April 2020)

*Lives are more important than political careers so we should rely on facts and defer to the experts*.

(*Sunday Independent*, 19 April 2020)

Suggestions of political leaders ignoring scientific advice attracted criticism. In ROI media, this usually related to other jurisdictions: Ireland’s regard for scientific advice was contrasted favourably with other countries such as the UK, who were presented as insufficiently science-led in their pandemic responses.

*Where countries on either side of us have flip-flopped and changed tack with astonishing rapidity, the Irish authorities have followed a graduated approach based on the best available international and local advice. In other countries, some politicians have played politics with the pandemic, but in the State the lead has been taken by scientific experts, with the Government effectively playing a support role that has demanded the provision of large sums of money*.

(*Irish Times*, 26 March 2020)

I *would like to see the science behind this decision. The stats and projections from other countries make me question these suggestions from Boris Johnson*

(*Tweet*, 13 March 2020)

However, agreement that scientific advice should determine policy decisions was not universal. As the pandemic developed, there emerged a dialectical narrative that positioned science and economics as contradictory forces. Here, ‘science’ was conflated with exclusively valuing preservation of life, whereas ‘economics’ represented broader concerns about the prosperity of society and mental wellbeing.

*Given the scale of economic carnage and human misery coming over the hill, every arm of the State should be on a war footing to save small businesses. Instead, those in tourism and retail are left exposed on the front line as cannon fodder, as Ireland pursues an economically-reckless scientific experiment to throttle a virus that other European countries are learning to keep at bay. […] If the medics’ iron-clad influence over national decision-making is not counter-balanced from here on in by pragmatic and assertive economic voices, we risk a deep, self-inflicted gash in society and the economy*.

(*Irish Times*, 29 May 2020)

*The coronavirus lockdown is an act of unprecedented public restriction, on an unparalleled scale. The government’s scientists say it is absolutely necessary in order to save lives, and to avoid the NHS collapsing under the strain of too many seriously ill patients. But the consequences of this vast social experiment—for people’s lives, their livelihoods, their liberty, their mental and physical health—are enormous, and radically unpredictable*.

(*Belfast Telegraph*, 3 April 2020)

Nowhere in the data was the validity of the science vs. economics dichotomy challenged. Instead, the dilemma was to be mediated by politics, which was ascribed the task of balancing the apparently competing priorities.

*[…] the current crisis engages profound ethical and moral dilemmas—because it involves weighing a set of competing imperatives, including public health, unemployment, mental health and social solidarity. These are the ultimate political decisions*.

(*Irish Times*, 24 April 2020)

*If science had its way the lockdown would extend indefinitely until there was a vaccine or a treatment for COVID-19. But the political reality is that treating the country as if it were a giant hospital won’t work in the long term*.

(*Irish Examiner*, 30 April 2020)

#### 3.3.3. Science as Rhetoric

While the principle of evidence-based policy was largely viewed positively within the data, there was some suspicion that the role of science in policy decisions was more symbolic than substantive. Aspects of the data revealed concern that the rhetorical authority of science was invoked to both justify and delegitimise political decisions.

*Be wary of the politician who responds to criticism by claiming to be following the science. Just as generations of public figures have learned they can evade scrutiny or shut down a discussion simply by invoking legal advice or citing (invariably unspecified) constitutional obstacles, many world leaders have co-opted “the science” as a rhetorical shield against impertinent questions about their handling of the COVID-19 pandemic*.

(*Irish Times*, 18 April 2020)

*Can’t help feeling we’re being hoodwinked when the government uses the term #ScienceLedAction—scientific endeavour involves airing alternative views to reach consensus and maybe there is no actual consensus on tacking the pandemic*.

(*Tweet*, 19 May 2020)

On the island of Ireland, the rhetorical functions of appeals to science were highlighted by exposure to differing policy decisions and rationales in the two jurisdictions on the landmass. Observation that NI/UK and ROI political leaders both claimed to derive policy from scientific advice, yet adopted differing policies, raised suspicions about selective or biased invocation of scientific evidence.

*While everyone says they are following the science, one can’t help wondering whether Northern Ireland’s historic divisions even extend to coronavirus. Who should we follow: London or Dublin*?

(*Irish Independent*, 14 March 2020)

*At least he’s no longer hiding behind the catchphrase of March—“we’re following the science”—since clearly the British government wasn’t. Though even then Swann [NI Minister of Health], under pressure to adopt an all-Ireland approach, came out with this stunner: “We’re following the science as it applies to Northern Ireland.” So chemistry is different in Monaghan and Cavan*?

(*Irish News*, 27 May 2020)

*Looks like UK Government are going for herd immunity for #COVID_19. Expecting 60% of the population to get the virus strikes me as a major gamble with people’s lives. Scientists across the world clearly differ*.

(*Tweet*, 13 March 2020)

Further acknowledgement of the politicisation of science involved allusions to suppression of politically damaging scientific results; however, this was only mentioned in the context of other countries, notably China and the USA.

*Scientific research is also being suppressed. According to a later report by the independent Caixin news agency, ‘an employee of one genomics company received a phone call from an official at the Hubei Provincial Health Commission, ordering the company to stop testing samples from Wuhan related to the new disease and destroy all existing samples... They were told to cease releasing test results, and report any future results to authorities.’*

(*Irish Daily Mail*, 20 April 2020)

*Aside from COVID-19, another thing the US government got from China is political control of scientists. Congress needs to stand up for science and stop undermining the exchange of ideas within the scientific community*.

(*Tweet*, 28 February 2020)

## 4. Discussion

COVID-19 is arguably the most important science communication challenge of a generation, yet follows a posited populist turn against scientific expertise in western societies. The current study helps to enlighten public orientations to science during the COVID-19 pandemic, by exploring the position afforded to science in news and social media coverage of COVID-19 in Ireland. Although exploratory and descriptive, the analysis raises some tentative suggestions for science communicators during the pandemic: for example showing how the scientific enterprise can be humanized by describing the day-to-day work by diverse actors that generates scientific insights; the utility of contextualizing cases of scientific uncertainty or dissensus within accessible explanations of the philosophy and process of science; the need to avoid positioning science as competing with other societal values, such as economic progress; and the importance of avoiding ‘deficit model’ communications that disparage lay concerns. The study suggests that COVID-19 raises both opportunities and risks for the relationship between science and society.

The analysis showed that representations of science in both news and social media were largely positive. Science was invested with a complex set of emotions including admiration, gratitude and hope. Some media articles explicitly contrasted this apparently revitalised public appreciation of science with a perceived cultural disenchantment from science in recent times. However, it should be noted that the media associated this discontent predominantly with other countries, notably the US and Great Britain. There are no empirical data indicating declining public confidence in science was underway in Ireland prior to the pandemic [27]. Thus, this analysis does not function as a ‘test’ of the proposition that COVID-19 has re-ignited societal trust in science, but rather to illustrate how the pandemic offers a platform for expressions of public support for science. These often extended beyond praise of specific coronavirus research efforts, to global endorsements of the process or philosophy of science. This supports suggestions that COVID-19 may boost public valuation of science, raising the potential for spillover effects to other societal challenges such as climate change. 

The few negative responses to science evident in the data predominantly involved frustration with contradictory or unclear scientific communications. The data did not reveal evidence of overt rejection or resistance of science. Despite previous concerns regarding the risks of misinformation about COVID-19, particularly on social media [38,44,46,47,48,49], this did not materialise in this corpus. Endorsement of specific ‘conspiracy theories’ about COVID-19′s origins was extremely rare, with such ideas primarily mentioned in the context of their refutation. This contrasts with previous indications that about half of UK and US adults have been exposed to COVID-19 misinformation [67,68]. It is possible that ‘conspiracy theories’ may be less prevalent in the Irish media environment. Alternatively, such content may be primarily transmitted via networks not sampled in this study (e.g., private Twitter messages or other platforms such as WhatsApp) or preferentially directed towards select user groups by social media algorithms. It is also possible that selection of articles and tweets that included the word-stem “scien*” may have biased the sample against such content. Furthermore, data were collected before a viable COVID-19 vaccine became available: more vaccine-related misinformation may have since cohered [69] (although at time of writing, ROI has one of the highest rates of vaccine uptake in the EU [70]). These caveats notwithstanding, the findings do align with recent studies reporting that reliable science-based information outweighs the prevalence of misinformation in social media coverage of COVID-19 [43,44].

Cultural concerns about COVID-19 misinformation can express a deficit model of science communication, in their censure of scientifically erroneous lay understandings. Statements that disparaged the contributions of non-scientists to debates about COVID-19 science and policy were evident in the data, particularly in social media. This is consistent with evidence that despite explicit endorsement of dialogical approaches to communication [71], deficit model assumptions persist among both scientists [72,73] and laypeople [74]. Importantly, rejecting deficit model approaches to COVID-19 conspiracy theories does not require deeming these beliefs legitimate: they can still be denounced on pragmatic grounds due to their links with reduced protective behaviour, intergroup prejudice and violence [38,75]. Yet rather than demonising or belittling those who endorse fringe beliefs about COVID-19, a more productive research and engagement strategy may involve exploring the social and psychological functions that those beliefs serve, which include ideologies, identities and fears [53,75,76,77]. While the data showed acknowledgement that science could provoke positive emotions such as hope, the analysis did not reveal any media attempts to engage with the emotional experiences that may motivate people to dispute scientific evidence.

During the pandemic, science coverage afforded real-time insight into the evolution of scientific understanding of a unique and unforeseen phenomenon. Perhaps inevitably, this drew public attention to instances of scientific disagreement or changes in scientific advice, for example regarding face-coverings [52]. Scientific inconsistency may be especially salient in Ireland, where pandemic responses are influenced by three governments (ROI, UK, and the devolved NI Executive), whose policy decisions and rationales differed at specific points during the pandemic [62]. Recent experimental research indicates media coverage of scientific uncertainty may be a double-edged sword [78]. Downplaying uncertainty in COVID-19 modelling yields immediate gains for public trust in science and support for science-based policy; however, this risks longer-term damage to trust if projections ultimately prove inaccurate [78]. In this analysis, observation of inconsistency in scientific statements did cause immediate frustration in both news and social media. However, this was tempered by numerous media articles that used the pandemic to illustrate how the philosophy of science renders all scientific knowledge provisional and subject to change. This coheres with Kreps and Kriner’s [78] recommendation that explaining and contextualising the uncertainty inherent in scientific knowledge represents the most strategic approach for promoting sustainable public confidence. COVID-19 may offer an opening to raise awareness of scientific uncertainty [79] and specific quality-control processes such as peer review [17] or randomised controlled trials [80]. 

Beyond abstract issues of scientific epistemology, the pandemic also offered the opportunity to raise awareness of the daily realities of scientific research. Previous research suggests lay alienation from science is reinforced by stereotypical media portrayals of scientists, which emphasise men with white hair and lab-coats who display obsession, eccentricity and social awkwardness [81,82,83,84,85]. This analysis showed that COVID-19 coverage emphasised the diversity of scientific actors, who encompass lab technicians and pharmaceutical employees as well as university scientists. Personalised profiles of individual scientists may humanise the scientific enterprise, although possibly also risk undermining understanding of science as a collective, team-based enterprise. It should be noted that most of the scientific spokespeople featured in the media were white males, which may not assist drives to increase the representation of women or ethnic minorities in STEM disciplines [86,87].

A final risk for science communication during the pandemic is the positioning of science in opposition to economic prosperity and community wellbeing. Nowhere in the data was the simplistic science vs. economics dichotomy challenged by observing that a raging pandemic threatened economic progress or that economic disadvantage affects public health. The positioning of politics as the mediator between scientific and economic interests may heighten concerns about the political co-option of science. Media showed sensitivity to the rhetorical functions of science, which was used to justify contradictory political positions during the pandemic. Heightened sensitivity to the politicisation of science may compromise public trust [88]. However, it could also be argued that public awareness of the selective recruitment of science to serve political interests signals an appropriately critical populace in an international context where COVID-19 science is indeed being widely politicised [18,57,78].

### Strengths and Limitations

The contributions of this study should be considered in light of its methodological strengths and limitations. The dataset included a range of news outlets from both ROI and NI, which were complemented by contemporaneous content from a popular social media platform. Media data offer assurance that the ideas analysed have been produced and consumed organically, independently of any preconceived research agenda. However, there is no direct correspondence between media content and public thinking [33,34]. A recent UK study found only half of respondents trusted media coverage of science, with online sources particularly distrusted [12]. Moreover, public reception of scientific messages hinges on their match with abiding cultural value-systems [89,90]. As the media content analysed in this study could be ignored, disputed or reinterpreted by audiences, the analysis does not claim to reflect public attitudes or beliefs. Direct research with members of the public is required to confirm the effects of exposure to this media coverage [78].

Further limitations pertain to the parameters of the datasets. Both data collection strategies relied on selection of specific keywords, which may have biased the samples. The Twitter data were restricted to tweets from public accounts with geolocation enabled; while necessary for ethical and logistical reasons, this restricted the number of tweets available and content from such accounts may not be representative of the entire population of Twitter users. Moreover, other social media networks, such as Facebook and WhatsApp, are more used in Ireland [61]. Although logistically difficult for researchers to access, content from such networks would complement the current dataset. A further study limitation was the lack of data on the sources of the tweets, which made it difficult to discern potential inter-relationships between data units—for example, if different tweets occurred in the same Twitter thread, or whether some tweets were published by social media accounts of the newspapers included in the analysis. A smaller-scale analysis, which tracks the public reception of specific scientific news stories, may yield useful granular detail on communicative dynamics within and between media sources [91]. Alternatively, a ‘big data’ approach could identify disparities between different media platforms; for example, news media may depart from social media’s documented tendency to focus on short-term over long-term implications of the pandemic [92].

Study conclusions are also limited by its lack of baseline data: direct comparison between pre- and post-pandemic media content is necessary to isolate the longitudinal effects of COVID-19. Furthermore, the analysis is restricted to one small region where science is underfunded (World Bank, 2019) and additional research is required to establish international comparability. The current datasets may prove a useful exemplar of responses to COVID-19 in a region where, compared with many other jurisdictions, science is more trusted [28], the media landscape less polarised [60], and pandemic responses less politicised [93,94]. Studies of other countries that entered the pandemic with more precarious trust in science may yield divergent results.

## 5. Conclusions

Science invites a multifaceted compound of responses in contemporary society. On the one hand, it is valorised as a critical source of cultural authority, with the appellation of ‘evidence-based’ functioning to flag the legitimacy of a policy, product or opinion. On the other, science is seen as a socially distant domain, with large segments of the public ready to demur from scientific consensus on politicised issues such as climate change or vaccines [95]. The current analysis suggests that in the Irish context, COVID-19 has functioned to cultivate appreciation of the value, applications, process and practice of scientific research. The pandemic offered a platform to make a compelling case for the relevance and practical importance of science generally and specific research fields. However, those interested in this opportunity to promote positive science–society relationships should also be aware of several risks, including feeding public alienation by purveying deficit model assumptions, reinforcing stereotypical images of scientists, and intensifying the politicisation of scientific statements.

## Figures and Tables

**Table 1:** Frequency of articles from the newspapers included in the analysis.

Jurisdiction	Newspaper	Number of Articles
Republic of Ireland	*The Irish Times*	261
	*Irish Independent*	146
	*Irish Daily Mail*	108
	*Sunday Independent*	69
	*Irish Examiner*	59
	*The Herald*	10
	TOTAL	653
Northern Ireland	*Belfast Telegraph*	215
	*Irish News*	60
	*Sunday Life*	14
	*Impartial Reporter*	8
	*News Letter*	2
	TOTAL	299

**Table 2:** Frequency of tweets from the Republic of Ireland and Northern Ireland.

Jurisdiction	Number of Articles
Republic of Ireland	446
Northern Ireland	157
TOTAL	603

## Data Availability

All experimental data to support the findings of this study are available upon request from the corresponding author.
